# Resveratrol Butyrate Esters Reduce Hypertension in a Juvenile Rat Model of Chronic Kidney Disease Exacerbated by Microplastics

**DOI:** 10.3390/nu16234076

**Published:** 2024-11-27

**Authors:** Yi-Ning Huang, Chien-Ning Hsu, Chih-Yao Hou, Shin-Yu Chen, You-Lin Tain

**Affiliations:** 1 Department of Pediatrics, Kaohsiung Chang Gung Memorial Hospital, Kaohsiung 833, Taiwan; b0102029@cgmh.org.tw; 2 Department of Pharmacy, Kaohsiung Chang Gung Memorial Hospital, Kaohsiung 833, Taiwan; cnhsu@cgmh.org.tw; 3 School of Pharmacy, Kaohsiung Medical University, Kaohsiung 807, Taiwan; 4 Department of Seafood Science, National Kaohsiung University of Science and Technology, Kaohsiung 811, Taiwan; chihyaohou@webmail.nkmu.edu.tw; 5 Department of Food Science, National Pingtung University of Science and Technology, Pingtung 912, Taiwan; 6 College of Medicine, Chang Gung University, Taoyuan 330, Taiwan; 7 Institute for Translational Research in Biomedicine, Kaohsiung Chang Gung Memorial Hospital, Kaohsiung 833, Taiwan

**Keywords:** resveratrol, hypertension, gut microbiota, kidney disease, microplastics, asymmetric dimethylarginine, butyrate renin–angiotensin system

## Abstract

Background: Resveratrol is recognized as a promising nutraceutical with antihypertensive and prebiotic properties; however, its bioavailability in vivo is limited. To enhance its bioactivity, we developed resveratrol butyrate esters (RBEs). This study investigates whether RBEs can mitigate hypertension induced by chronic kidney disease (CKD) and exacerbated by microplastics (MPs) exposure in juvenile rats. Methods: Three-week-old male Sprague Dawley rats were fed either regular chow or 0.5% adenine chow for three weeks. The adenine-fed CKD rats (N = 8 per group) received either 5 μM MPs (10 mg/L) or MPs combined with RBE (25 mg/L) in their drinking water from weeks 3 to 9. Results: Our results indicate that MP exposure worsened CKD-induced hypertension, while RBE treatment resulted in a reduction in systolic BP by 15 mmHg (155 ± 2 mmHg vs. 140 ± 1 mmHg, *p* < 0.05). The combined exposure to adenine and MPs was associated with nitric oxide (NO) deficiency, which RBE treatment alleviated. Additionally, our findings revealed that RBE modulated both the classical and nonclassical renin–angiotensin system (RAS), contributing to its protective effects. We also observed changes in gut microbiota composition, increased butyric acid levels, and elevated renal GPR41 expression associated with RBE treatment. Conclusions: In conclusion, in this juvenile rat model of combined CKD and MP exposure, RBE demonstrates antihypertensive effects by modulating NO levels, the RAS, gut microbiota, and their metabolites.

## 1. Introduction

Phenolic compounds are abundantly found in plants and exhibit a variety of biological effects, including antioxidant, anti-inflammatory, anti-atherosclerotic, and anti-aging properties [1,2]. Resveratrol, a natural stilbene phenolic compound, is commonly utilized as a nutraceutical for the prevention of cardiovascular disease (CVD), type 2 diabetes mellitus, chronic kidney disease (CKD), and various other health conditions [3,4,5]. Recent research underscores the importance of understanding resveratrol’s bioefficacy and bioavailability to elucidate its role in human health [6]. Like many phenolic compounds, resveratrol has low bioavailability [7], which can be influenced by several factors [8]. For example, interactions between resveratrol and gut microbiota may affect its biological effects [9]. Notably, the limited in vivo bioactivity of resveratrol poses a significant challenge for its clinical applications. Recent evidence suggests that the esterification of resveratrol could enhance its bioactivity [10,11].

Previously, we developed esterified resveratrol-producing short-chain fatty acid (SCFA) products through Steglich reactions to enhance the bioactivity of resveratrol [12]. These resveratrol–SCFA esters exhibited antioxidant properties, with resveratrol butyrate esters (RBEs) demonstrating biological activity comparable to that of resveratrol but with improved bioavailability [13]. SCFAs, essential microbial metabolites, function as postbiotics and have been shown to positively influence human health [14,15].

In children, hypertension is often secondary to CKD, unlike in adults, where it is typically due to primary causes [16]. Despite treatment with antihypertensive medications, many children with CKD remain uncontrolled and may progress to end-stage kidney disease. Furthermore, CKD-related morbidity can be worsened by environmental pollutants, such as microplastics (MPs) [17]. Although research on the impact of MPs on kidney function is still in its early stages, there is growing concern that CKD patients may be especially vulnerable to the accumulation and damaging effects of these pollutants [18].

Resveratrol has demonstrated antihypertensive and renoprotective effects in several animal models [19], including those involving prenatal hypoxia combined with high-fat intake [20], spontaneously hypertensive rats [21], and a renovascular hypertensive two-kidney one-clip model [22]. However, these benefits have not consistently translated into positive outcomes in human populations. A meta-analysis of 10 randomized controlled trials found no significant effect of resveratrol supplementation on cardiovascular risk factors [23]. Another meta-analysis, which included 32 studies, suggested a mild renal protective effect of resveratrol in adults, though it was based on low-certainty evidence [24].

In our previous study using an adenine-induced juvenile rat model of CKD, we found that RBE supplementation improved kidney function and reduced hypertension by modulating the gut microbiota [25]. The beneficial effects of RBE on blood pressure (BP) are thought to stem from its ability to regulate key factors such as vasodilators, including nitric oxide (NO), and vasoconstrictors within the renin–angiotensin system (RAS). Building on this, the goal of our current study is to investigate whether exposure to MPs exacerbates hypertension in juvenile rats with adenine-induced CKD. Additionally, we aim to determine whether RBE treatment can mitigate these effects by influencing the gut microbiota, the NO signaling pathway, and the RAS.

## 2. Materials and Methods

### 2.1. Synthesis of Resveratrol Butyrate Esters

The RBEs were synthesized following our established protocol [11]. Initially, butyric acid (ACROS, Morris Plains, NJ, USA) was combined with resveratrol (TCI Development Co., Ltd., Shanghai, China) in tetrahydrofuran (Echo Chemical Co., Ltd., Miaoli County, Taiwan). Subsequently, 4-dimethylaminopyridine (Sigma-Aldrich, Saint Louis, MO, USA) and N-ethyl-N-(3-dimethylaminopropyl) carbodiimide (Sigma-Aldrich) were added to the mixture. The esterification reaction was conducted in the absence of light for 48 h. After the reaction was complete, the mixture was diluted with distilled water and filtered to isolate the precipitated RBEs. The final RBEs were then stored in a freezer at −20 °C for preservation.

### 2.2. Animals

The experimental protocol received approval from the Institutional Animal Ethics Committee (permit number: 2024022301, approval date: 27 March 2024). Sprague Dawley rats were allowed to acclimate in a temperature-controlled room (22 ± 1 °C), with 55 ± 5% humidity and a 12:12 light–dark cycle, in an AAALAC-International accredited animal facility. Pregnant rats were provided with unrestricted access to food and water until delivery, and the offspring were raised alongside their dams during the lactation period.

After weaning, two rats were housed per cage. Male rats aged three weeks were divided into two dietary groups: one group received regular chow, while the other received chow containing 0.5% adenine for three weeks to induce CKD. Only male juvenile rats were used due to their heightened susceptibility to developing hypertension at an earlier age compared to females [26]. The adenine dosage was selected based on our previously established pediatric CKD model [25]. Some of the rats were given MPs in their drinking water from week three to nine. The polystyrene MPs were procured from Magsphere (Pasadena, CA, USA), and the dosages of MPs and resveratrol were determined based on prior studies involving rats [27].

**Figure 1:**
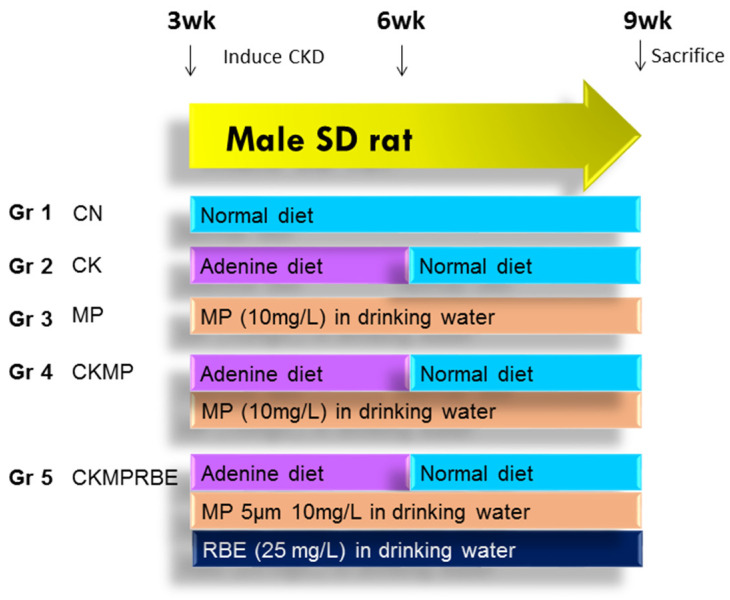
Experimental protocol employed in the current study.

Figure 1 demonstrates the experimental protocol. The male rats were randomly assigned to five groups (n = 8 per group): a control group (CN), a CKD group receiving adenine chow (CK), an MP group receiving 5 μM microparticles (MPs) (10 mg/L), a CKD + MP group (CKMP) receiving both adenine and MPs, and a CKD + MP + RBE group (CKMPRBE) receiving adenine, MPs, and RBE at a dose of 25 mg/L in drinking water from weeks three to nine, as detailed in our previous work [25].

Non-invasive BP measurements were conducted on conscious, trained rats using the tail-cuff method (CODA, Kent Scientific Corp., Torrington, CT, USA). The rats were acclimatized to the restraint holders for one week prior to the recording sessions. Three consistent BP measurements were taken, and the average value was calculated. All rats were euthanized at nine weeks of age. Fecal samples were collected in the morning, before sacrifice, by gently lifting the tail and twisting it toward the back to induce defecation. The fecal samples were then stored at −80 °C until further extraction. The rats were anesthetized by an intraperitoneal injection of ketamine (50 mg/kg) and xylazine (10 mg/kg) and euthanized with an intraperitoneal injection of a pentobarbital (150 mg/kg) overdose. Both kidneys were excised and weighed, and plasma samples were collected, aliquoted, and stored at −80 °C.

### 2.3. Gut Microbiota Metagenomics

Microbial DNA was extracted from stool samples, and metagenomic analysis was performed using 16S ribosomal RNA genes [25]. The barcode primers designed for SMRTbell library preparation (Menlo Park, CA, USA) were employed to amplify the full-length 16S rRNA gene from the genomic DNA. We conducted downstream analyses of the sequences using the QIIME2 software package [28]. From the amplicon sequence variants (ASVs), we constructed a phylogenetic tree with FastTree version 2.1.10, and each cluster was taxonomically assigned. For α-diversity analysis, we assessed observed features and calculated the Shannon index. β-diversity was evaluated using the analysis of similarities (ANOSIM) in conjunction with Partial Least Squares Discriminant Analysis (PLSDA). We also utilized the linear discriminant analysis (LDA) effect size to identify bacterial taxa with significantly different abundances between the groups [29]. To assess statistical differences between the groups, we conducted a two-sided Welch’s *t*-test using the Statistical Analysis of Metagenomic Profiles (STAMP).

### 2.4. Determination of Plasma SCFAs

We assessed SCFA concentrations in plasma using a Gas Chromatograph–Mass Spectrometer (Agilent Technologies, Santa Clara, CA, USA), following previously established methods [25]. The SCFAs analyzed included acetic acid, propionic acid, butyric acid, isobutyric acid, isovaleric acid, and valeric acid. Chromatographic separation was achieved using a DB-FFAP column (30 cm × 0.25 mm, 0.25 µm; Agilent Technologies), with 2-ethylbutyric acid serving as the internal standard. A 1 µL injection volume was applied with a split ratio of 5:1 at an injection temperature of 240 °C.

### 2.5. Quantitative PCR

Total RNA was extracted from kidney cortex samples, followed by real-time quantitative PCR (qPCR), as previously described in our methods [25]. We analyzed the expression of SCFA receptors [30] and components of the RAS [31]. All samples were analyzed in duplicate, with 18S ribosomal RNA (18S rRNA) serving as the reference gene for normalizing the qPCR data. The comparative threshold cycle (Ct) method was employed for the relative quantification of gene expression. The primer sequences are listed in Table 1.

### 2.6. Measurement of NO-Related Parameters

Plasma levels of L-arginine and asymmetric dimethylarginine (ADMA), an inhibitor of nitric oxide synthase, were measured using high-performance liquid chromatography (HP Series 1100, Agilent Technologies, Inc., Santa Clara, CA, USA) with o-phthalaldehyde and 3-mercaptopropionic acid as the derivatization reagents [25]. L-homoarginine (Fluka, Neu Ulm, Germany) was used as the internal standard.

### 2.7. Statistical Analysis

Data are presented as the mean ± standard error of the mean (SEM). The data were analyzed using a one-way analysis of variance (ANOVA) followed by multiple comparisons with Tukey’s post hoc test. A *p*-value of less than 0.05 was considered statistically significant for all tests. The analysis was conducted using SPSS version 17.0 software.

## 3. Results

### 3.1. Anthropometrics and Blood Pressure

**Figure 2:**
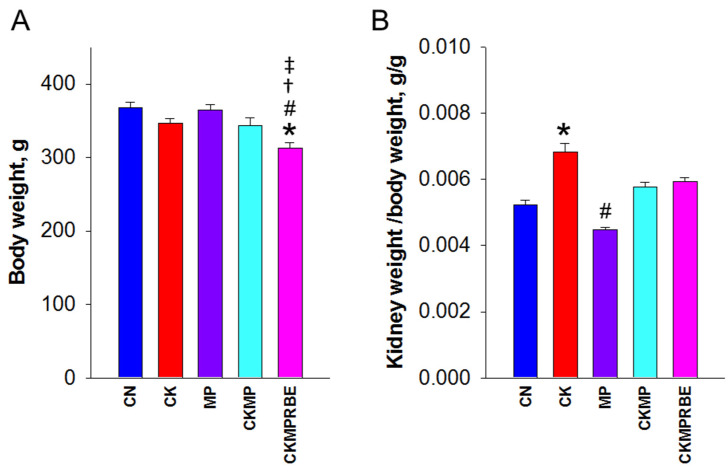

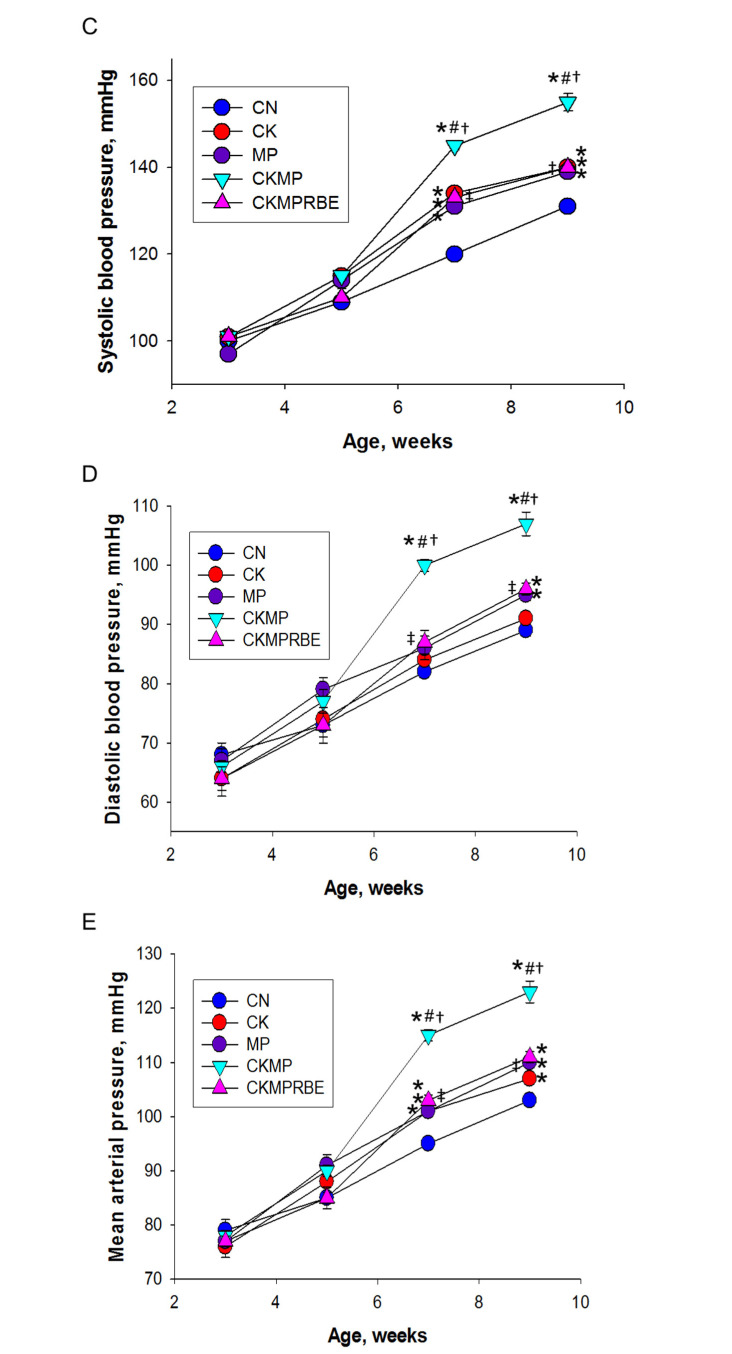
(**A**) Body weight, (**B**) kidney weight-to-body weight ratio, (**C**) systolic blood pressure, (**D**) diastolic blood pressure, and (**E**) mean arterial pressure in rats receiving adenine chow (CK), microplastics (MP), or resveratrol butyrate esters (RBE). N = 8/group; * *p* < 0.05, compared with rats fed with regular chow (CN), # *p* < 0.05, compared with rats fed with adenine chow (CK); † *p* < 0.05, compared with rats exposed to microplastics (MP); and ‡ *p* < 0.05, compared with rats fed adenine chow and exposed to MP (CKMP).

No mortality was observed among the five groups at nine weeks of age. The CKMPRBE group exhibited the lowest body weight (BW) (Figure 2A). The kidney weight (KW)-to-BW ratio was higher in the CK group compared to the CN and MP groups (Figure 2B). After seven weeks, adenine-exposed and MP-exposed juvenile rats demonstrated elevated systolic BP compared to the CN group (CK group: 134 ± 1 mmHg, MP group: 131 ± 1 mmHg, vs. CN group: 120 ± 1 mmHg, both *p* < 0.05), with a synergistic effect observed from their combined exposures (CKMP group: 145 ± 1 mmHg, *p* < 0.05) (Figure 2C). Importantly, the increase in systolic BP in the CKMP group was mitigated by RBE treatment (CKMPRBE group: 133 ± 1 mmHg, *p* < 0.05). At week 9, MP exposure exacerbated CKD-induced hypertension (CKMP group vs. CK group: 155 ± 2 mmHg vs. 140 ± 1 mmHg, *p* < 0.05), while RBE treatment reduced systolic BP by 15 mmHg (CKMPRBE group: 140 ± 1 mmHg, *p* < 0.05). Diastolic BP (Figure 2D) and mean arterial pressure (Figure 2E) showed similar trends as systolic BP.

### 3.2. Plasma SCFAs and SCFA Receptors

Adenine and MP exposure differentially affected plasma concentrations of SCFAs. Adenine exposure resulted in lower plasma levels of isobutyric acid (*p* = 0.042) and valeric acid (*p* = 0.02) in the CK group, while MP exposure led to reduced propionic acid levels in the MP group (*p* = 0.004) compared to the CN group (Table 2). Notably, combined adenine and MP exposures did not impact plasma SCFA concentrations. In contrast, RBE significantly increased butyric acid levels in the CKMPRBE group compared to the CKMP group (*p* = 0.002). Plasma concentrations of isovaleric acid were consistent across all five groups, showing no significant differences.

**Figure 3:**
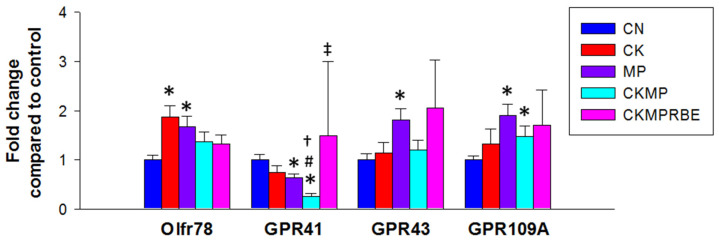
Renal mRNA expression of olfactory receptor 78 (OlfR78); G protein-coupled receptor 41 (GPR41), GPR43, and GPR109A in rats receiving adenine chow (CK), microplastics (MPs), or resveratrol butyrate esters (RBEs). N = 8/group. * *p* < 0.05, compared with rats fed with regular chow (CN); # *p* < 0.05, compared with rats fed with adenine chow (CK); † *p* < 0.05, compared with rats exposed to microplastics (MPs); and ‡ *p* < 0.05, compared with rats fed adenine chow and exposed to MP (CKMP).

SCFAs can influence BP by interacting with SCFA receptors [30]. We analyzed the expression of four SCFA receptors, containing olfactory receptor 78 (OlfR78), G protein-coupled receptors 41 (GPR41), GPR43, and GPR109A. Figure 3 shows that renal Olfr78 expression was significantly higher in the CK and MP groups compared to the CN group. Additionally, combined adenine and MP exposures resulted in the lowest GPR41 expression in the CKMP group, but this reduction was restored with RBE treatment. No significant differences were observed in GPR43 and GPR109A expression between the CN and CK groups; however, both were induced by MP exposure.

### 3.3. Gut Microbiota Composition

**Figure 4:**
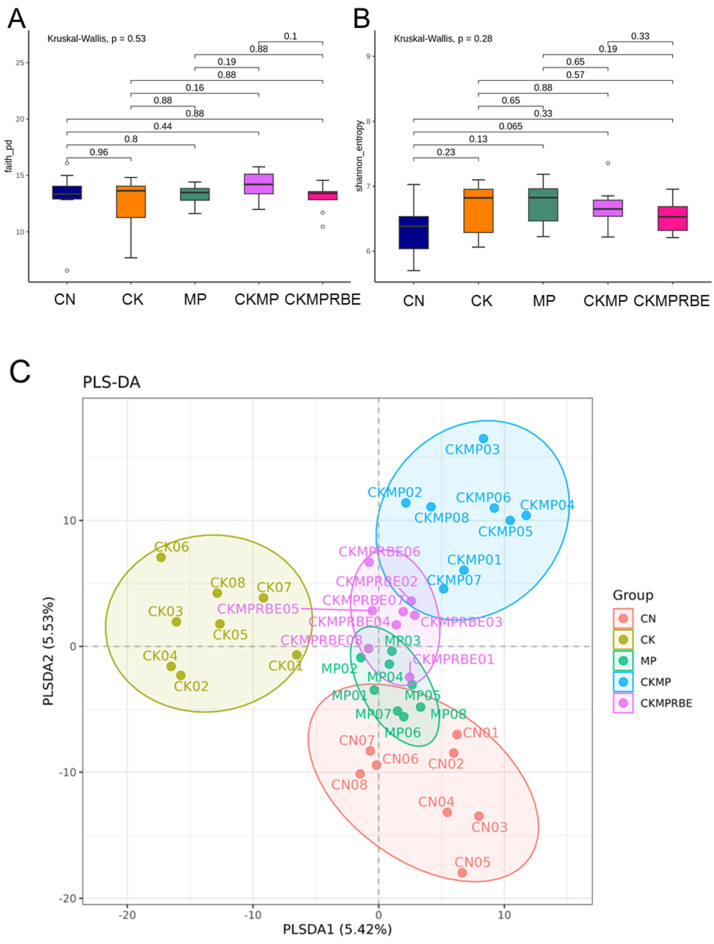
For α-diversity analysis among the groups, we utilized (**A**) the Faith’s Phylogenetic Diversity (PD) index and (**B**) the Shannon index. For the β-diversity assessment, we performed (**C**) Partial Least Squares Discriminant Analysis (PLSDA). Each point represents the microbiota of an individual sample, with colors indicating the corresponding group. N = 8 per group. CN, rats fed with regular chow; CK, rats fed with adenine chow; MP, rats exposed to microplastics; CKMP, rats fed adenine chow and exposed to MP; and CKMPRBE, rats fed adenine chow and exposed to both MP and resveratrol butyrate esters.

Both α-diversity metrics—the Faith’s PD index and the Shannon index—assessing microbiome richness and evenness showed no significant differences among the five groups (Figure 4A,B). Additionally, β-diversity was visualized through a PLSDA plot, which revealed five distinct clusters (Figure 4C). ANOSIM analysis confirmed significant differences among nearly all the groups (all *p* < 0.05), except for the comparison between the CN and MP groups, which did not reach statistical significance.

**Figure 5:**
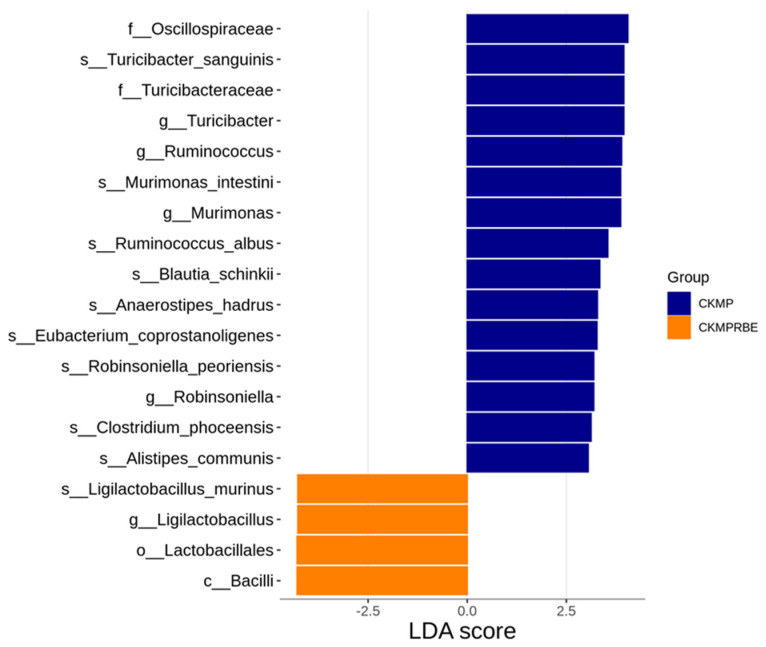
Linear discriminant effect size (LEfSe) analysis revealed significant differences in bacterial taxa between the CKMP (blue) and CKMPRBE (orange) groups, with LDA scores exceeding 3.0. CKMP, rats fed adenine chow and exposed to MP; CKMPRBE, rats fed adenine chow and exposed to both MP and resveratrol butyrate esters.

The LEfSe analysis revealed significant differences in taxa abundance between the CKMP and CKMPRBE groups, as illustrated in Figure 5. Notably, *Turicibacter sanguinis*, along with its associated genus and family, was more abundant in the CKMP group (LDA > 3). Additionally, the genera *Ruminococcus*, *Murimonas*, and *Robinsoniella* were also enriched in the CKMP group. In contrast, the CKMPRBE group exhibited an increase in *Ligilactobacillus murinus*, along with its corresponding genus, order, and class, compared to the CKMP group.

**Figure 6:**
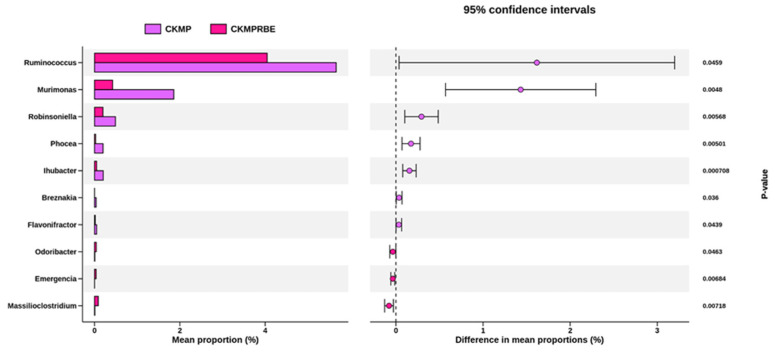
Differentially abundant taxa between the CKMP and CKMPRBE groups as identified by STAMP analysis with 95% confidence intervals. N = 8/group. CKMP, rats fed adenine chow and exposed to MP; CKMPRBE, rats fed adenine chow and exposed to both MP and resveratrol butyrate esters.

To identify the differentially altered taxa at the genus level due to RBE treatment, we applied the two-sided Welch’s *t*-test in STAMP to establish statistical differences. Compared to the CKMP group, RBE treatment resulted in significant changes in microbial genera, including notable increases in *Ruminococcus*, *Murimonas*, *Robinsoniella*, *Phocea*, *Ihubacter*, *Breznakia*, and *Flavonifractor*, as well as decreases in *Odoribacter*, *Emergencia*, and *Massilioclostridium* (Figure 6).

### 3.4. NO and RAS

**Figure 7:**
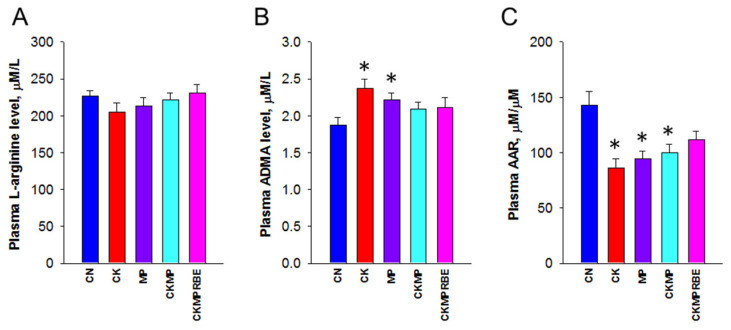
Plasma concentrations of (**A**) L-arginine, (**B**) asymmetric dimethylarginine (ADMA), and (**C**) their combined ratio (AAR) in rats receiving adenine chow (CK), microplastics (MPs), or resveratrol butyrate esters (RBEs). N = 8/group; * *p* < 0.05, compared with rats fed with regular chow (CN).

We next assessed NO and RAS as they play crucial roles in regulating BP. NO is a potent vasodilator, with L-arginine serving as the substrate for NO synthase to produce NO, while ADMA inhibits NO production. Our results showed that plasma concentrations of L-arginine were similar across all five groups (Figure 7A). Exposure to either adenine or MP resulted in increased plasma concentrations of ADMA (Figure 7B), but there was no synergistic effect between the two. The ratio of L-arginine to ADMA, which reflects NO bioavailability [32], was diminished by adenine, MP, or their combined exposures; however, this reduction was partially reversal by the RBE treatment (Figure 7C).

**Figure 8:**
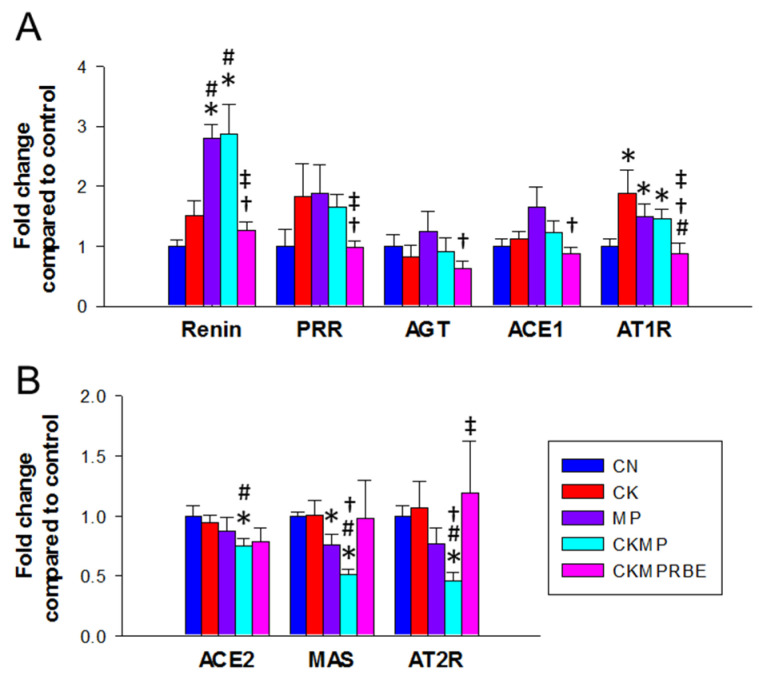
Renal mRNA expression of RAS-related genes in the (**A**) classical axis, which includes renin, pro(renin) receptor (PRR), angiotensinogen (AGT), ACE1, and AT1R; the (**B**) nonclassical axis, which includes ACE2, MAS, and AT2R in rats receiving adenine chow (CK), microplastics (MP), or resveratrol butyrate esters (RBE). N = 8/group. * *p* < 0.05, compared with rats fed with regular chow (CN); # *p* < 0.05, compared with rats fed with adenine chow (CK); † *p* < 0.05, compared with rats exposed to microplastics (MP); and ‡ *p* < 0.05, compared with rats fed adenine chow and exposed to MP (CKMP).

The RAS primarily raises BP through the action of angiotensin II (Ang II) as a potent vasoconstrictor. The binding of renin to the (pro)renin receptor (PRR) activates Ang-II-dependent and independent pathways to raise BP [31]. The classical RAS is characterized by the angiotensin-converting enzyme 1 (ACE1)-Angiotensin II type 1 receptor (AT1R) axis, which promotes vasoconstriction. In contrast, the nonclassical RAS, consisting of the ACE2-Ang-(1-7)-MAS axis and the Ang II/Ang III-AT2R pathway, counteracts the effects of the aberrant activation of the classical axis. We used qPCR to quantify changes in key RAS-related genes in rat kidneys. Figure 8 illustrates that exposure to adenine and MP tends to increase the expression of genes in the classical RAS axis, including renin and AT1R, while these increases were normalized by RBE treatment (Figure 8A). Compared to the CKMP group, RBE also reduced other components of the classical axis, such as PRR, AGT, and ACE1. Conversely, resveratrol treatment resulted in an increase in ACE2, MAS, and AT2R within the nonclassical RAS axis, which was inhibited by the combined exposure to adenine and MP (Figure 8B).

## 4. Discussion

In this study, we found that MP exposure exacerbated CKD-induced hypertension in young rats at nine weeks of age. Notably, we report for the first time that RBE can protect juvenile rats with CKD from hypertension exacerbated by MP exposure, resulting in a reduction in systolic BP by approximately 15 mmHg. Although the antihypertensive effect did not fully normalize BP, the 15 mm Hg reduction in systolic BP is considered clinically significant.

The key findings from our data are as follows: (1) juvenile rats exhibited heightened susceptibility to the detrimental effects of MPs, which exacerbated hypertension; (2) RBE treatments alleviated hypertension induced by the combined exposure to adenine and MP; (3) the beneficial effects of RBE were associated with increased levels of butyric acid and the elevated expression of renal GPR41; (4) combined adenine/MP-induced hypertension was linked to NO deficiency, which improved with RBE treatment; (5) RBE’s protective effect against hypertension in juvenile rats was correlated with a reduction in the renal expression of renin and AT1R in the classical RAS axis, along with an increase in ACE2, MAS, and AT2R in the nonclassical RAS axis; (6) combined exposure to adenine and MPs, as well as RBE treatment, differentially influenced distinct gut microbiota profiles; and (7) the protective effect of RBE was accompanied by alterations in several bacterial populations, including an increase in *Ligilactobacillus murinus* and a decrease in *Turicibacter sanguinis*.

In accordance with previous research on the antihypertensive effects of resveratrol [33,34], the treatment of young rats with RBE, a derivative of resveratrol, resulted in an approximate 15 mmHg reduction in SBP, effectively mitigating hypertension. The antihypertensive effects of RBE in the context of pediatric CKD align with our earlier report [25]. We expanded our investigation to explore its protective role in a juvenile rat model subjected to both adenine and MP exposure. Our study demonstrates that RBE protects young rats with CKD from hypertension exacerbated by MP exposure.

One beneficial effect of RBE on hypertension may be associated with improved NO bioavailability. Previous studies have indicated that NO deficiency is implicated in CKD and hypertension [35,36]. Conversely, the antihypertensive effects of resveratrol have been attributed to its ability to enhance NO levels [33]. The data support this notion, showing that RBE’s protective effect against hypertension aligns with an increased L-arginine to ADMA ratio, indicating enhanced NO bioavailability in the plasma.

Another protective effect of RBE is its regulation of the RAS. In aging mice, resveratrol has been shown to protect against arterial fibrosis by inhibiting the classical axis and stimulating the nonclassical axis [37]. In our young rat model of CKD-induced hypertension, RBE treatment decreased renin and AT1R levels in the classical RAS axis while increasing ACE2, MAS, and AT2R levels in the nonclassical axis. These findings suggest that RBE helps restore balance between the two RAS axes, promoting vasodilation and lowering BP.

Butyric acid is a crucial component of RBE and serves as a ligand for the SCFA receptors GPR41, GPR43, and GPR109A. It can influence BP by stimulating these receptors; GPR43 tends to elevate BP, while GPR41 and GPR109A can induce vasodilation. In our study, RBE treatment was found to increase plasma concentrations of butyric acid and the renal expression of GPR41 in the CKMPRBE group. These findings suggest that hypertension resulting from adenine and MP exposure may be mitigated by RBE’s modulation of SCFA receptors, shifting the balance from vasoconstriction toward vasodilation.

The protective mechanisms through which RBE treatment alleviates hypertension complicated by adenine and MP exposure are also associated with alterations in gut microbiota. Previous research indicates that the beneficial effects of resveratrol are closely linked to its prebiotic properties [38]. Although RBE treatment resulted in a distinct microbiome composition compared to the other groups, there was no significant difference in α-diversity among the five groups.

So far, data on the impact of MPs on the gut microbiota remain scarce [39]. A recent study revealed that MP exposure induces size-dependent multi-organ damage, including kidney injury in mice, accompanied by gut microbiota dysbiosis [40]. Although the gut-kidney axis plays a key role in CKD-induced hypertension [41], it remains unclear whether MPs contribute to CKD-induced hypertension through gut microbiota dysbiosis. Our findings indicate that MP exposure exacerbates CKD-induced hypertension, which is associated with an increase in *Turicibacter sanguinis* and its related genus and family. This aligns with previous research demonstrating that MPs can affect soil microbial communities, leading to an increase in the abundance of the genus *Turicibacter* [42]. *Turicibacter sanguinis* is the most predominant and well-studied species within the genus *Turicibacter*. This species has been identified in animal models with irritable bowel syndrome [43]. However, its potential role in the pathogenesis of hypertension has not yet been thoroughly investigated. We found that the species *Ligilactobacillus murinus*, along with its associated genus, order, and class, were enriched in the CKMPRBE group, correlating with the antihypertensive effects of RBE. This finding is consistent with previous research indicating that *Ligilactobacillus murinus* can have lower BP in spontaneously hypertensive rats [44].

In addition, STAMP analysis identified several bacterial taxa associated with hypertension, specifically the hypertension-depleted taxa *Ruminococcus*, *Robinsoniella*, and *Flavonifractor* [45]. Our findings indicated that the BP-lowering effect of RBE is correlated with an increase in the abundance of these taxa. Furthermore, RBE was found to decrease the proportion of the genus *Emergencia*, which is known to convert carnitine into trimethylamine N-oxide (TMAO) [46]. TMAO has been linked to the exacerbation of hypertension and kidney disease [47]. Further investigation is needed to determine if RBE’s protective effects against hypertension are linked to these specific microbial changes.

Our study has several limitations. First, using a single dose of RBE may not fully capture its effectiveness, and thus, further research with multiple dosing regimens is needed. Although we previously found that low-dose RBE (25 mg/L) and resveratrol (50 mg/L) similarly protected adenine-fed rats against hypertension and kidney damage [25], it remains unclear whether these protective effects are dose-dependent or may arise in a dose-independent manner, which warrants further investigation. Second, our focus on juvenile male rats highlights the need to explore potential sex differences in response to MPs and RBE. We concentrated RBE treatment on the CKMP group based on previous findings, but more research is required to assess RBE’s impact on BP in MP-exposed young rats. Lastly, RBE’s antihypertensive effects may involve other organs that regulate BP, necessitating further studies.

## 5. Conclusions

In conclusion, this study offers the first evidence that RBE may serve as a potential therapy targeting NO, the RAS, and gut microbiota to protect against hypertension induced by combined exposure to adenine and MP. Our findings provide novel mechanistic insights into the role of RBE in modulating these pathways and offer promising prospects for its application in managing hypertension. While our findings reveal novel mechanistic insights related to RBE, further investigation and clinical translation are essential to fully understand its therapeutic potential.

## Figures and Tables

**Table 1:** Primers for PCR amplification of each gene studied.

Gene	Sense	Anti-Sense
Olfr78	GAGGAAGCTCACTTTTGGTTTGG	CAGCTTCAATGTCCTTGTCACAG
GPR41	TCTTCACCACCGTCTATCTCAC	CACAAGTCCTGCCACCCTC
GPR43	CTGCCTGGGATCGTCTGTG	CATACCCTCGGCCTTCTGG
GPR109A	CGGTGGTCTACTATTTCTCC	CCCCTGGAATACTTCTGATT
Renin	AACATTACCAGGGCAACTTTCACT	ACCCCCTTCATGGTGATCTG
PRR	GAGGCAGTGACCCTCAACAT	CCCTCCTCACACAACAAGGT
AGT	GCCCAGGTCGCGATGAT	TGTACAAGATGCTGAGTGAGGCAA
ACE1	CACCGGCAAGGTCTGCTT	CTTGGCATAGTTTCGTGAGGAA
AT1R	GCTGGGCAACGAGTTTGTCT	CAGTCCTTCAGCTGGATCTTCA
ACE2	ACCCTTCTTACATCAGCCCTACTG	TGTCCAAAACCTACCCCACATAT
MAS	CATCTCTCCTCTCGGCTTTGTG	CCTCATCCGGAAGCAAAGG
AT2R	CAATCTGGCTGTGGCTGACTT	TGCACATCACAGGTCCAAAGA
R18S	GCCGCGGTAATTCCAGCTCCA	CCCGCCCGCTCCCAAGATC

**Table 2:** Plasma SCFA levels in rats receiving adenine chow (CK), microplastics (MPs), or resveratrol butyrate esters (RBEs).

Group	CN	CK	MP	CKMP	CKMPRBE
Acetic acid (μM)	1163 ± 53.9	1148.6 ± 55.1	1080.8 ± 26.1	1053.3 ± 47.5	966.2 ± 36.0 *#†
Propionic acid (μM)	6.0 ± 0.7	5.3 ± 0.5	3.4 ± 0.4 *#	4.8 ± 0.4 †	6.0 ± 0.5 †
Isobutyric acid (μM)	3.1 ± 0.3	2.4 ± 0.1 *	3.3 ± 0.3 #	2.6 ± 0.3	3.0 ± 0.4
Butyric acid (μM)	16.1 ± 0.9	18.4 ± 0.8	15.8 ± 0.7 #	17.6 ± 0.8	26.9 ± 2.3 *#†‡
Isovaleric acid (μM)	4.8 ± 0.4	5.4 ± 0.4	4.5 ± 0.2	5.2 ± 0.5	5.3 ± 0.3
Valeric acid (μM)	6.5 ± 0.2	4.9 ± 0.5 *	6.3 ± 0.2 #	5.7 ± 0.5	6.6 ± 0.3 #

N = 8/group. * *p* < 0.05, compared with rats fed with regular chow (CN); # *p* < 0.05, compared with rats fed with adenine chow (CK); † *p* < 0.05, compared with rats exposed to microplastics (MPs); ‡ *p* < 0.05, compared with rats fed adenine chow and exposed to MP (CKMP).

## Data Availability

Data are contained within the article.
